# Supplementary material for: Optomechanical synchronization across multi-octave frequency spans
Source: Nat Commun. 2021 Sep 24;12:5625. doi: 10.1038/s41467-021-25884-x (PMC8463541; doi:10.1038/s41467-021-25884-x)
Supplement: Supplementary file 1 — Supplementary Information [file 41467_2021_25884_MOESM1_ESM.pdf]

Supplementary Information:
Optomechanical Synchronization across Multi-Octave Frequency Spans

Caique C. Rodrigues[1,2,*] Cauê M. Kersul[1,2], André G. Primo[1,2], Michal Lipson[3,4], Thiago P. M. Alegre[1,2], and Gustavo S. Wiederhecker[1,2†]

[1] *Applied Physics Department, Gleb Wataghin Physics Institute, University of Campinas, Campinas, SP, Brazil*
[2] *Photonics Research Center, University of Campinas, Campinas, SP, Brazil*
[3] *Department of Electrical Engineering, Columbia University, New York, New York 10027, USA*
[4] *Department of Applied Physics and Applied Mathematics, Columbia University, New York, New York 10027, USA*

Supplementary Note 1.

Cavity Characterization. The whole experimental setup and the cavity geometry are shown in Supplementary Figure 1.

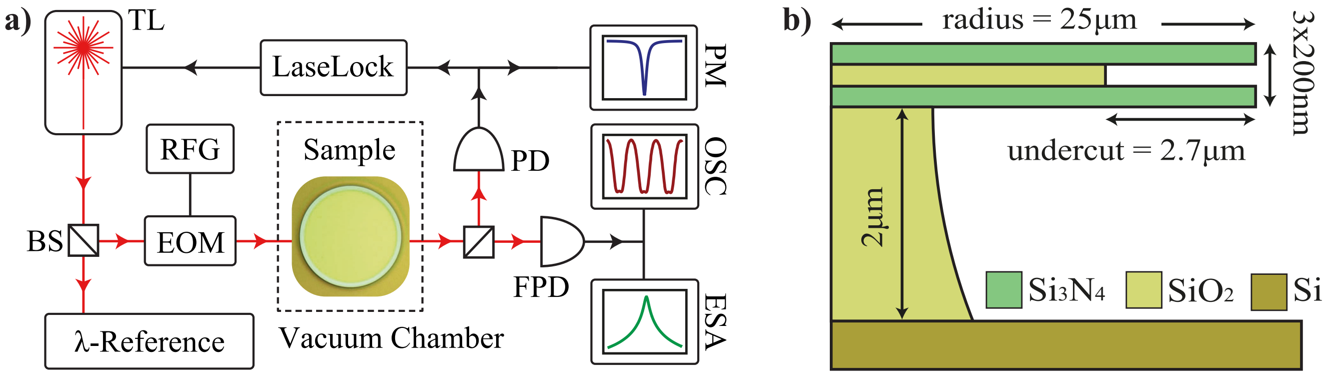

Supplementary Figure 1. **a)** Experimental setup used in the article. A tunable laser (TL) goes into a beam splitter (BS), in which one of the arms goes to a HCN cell wavelength reference, and the other arm goes to a electro-optical modulator (EOM) that is controlled by a radio frequency generator (RFG, Agilent PSG E8251). The modulated field after interacting with the sample inside a vacuum chamber of ≈ 0.1 mbar goes to another beam splitter which we finally obtain our results. The output signal then reaches a fast photodetector (FPD), which measures both the temporal trace using an oscilloscope (OSC, DSO9254A) and also the spectral content at a electrical spectrum analyzer (ESA, Keysight N9030), but also a slow photodetector (PD) which gives the Lorentzian shape optical transmission. The final part of the setup is a feedback loop (LaseLock) that goes back into the tunable laser that makes the laser wavelength stable by self referenciation, avoiding unwanted drifts during the data acquisition; **b)** Illustration of the nitride double disk cavity geometry used in the experiment.

The optical and the mechanical modes used in this experiment are shown in Supplementary Figure 2, with their best fits in red. The model of these curves are given by well-known Equation (1) and Equation (2)

$$T(\Delta) = \left|\frac{s_{\text{out}}}{s_{\text{in}}}\right|^2 = \frac{(1-2\eta)^2 + \frac{4\Delta^2}{\kappa^2}}{1+\frac{4\Delta^2}{\kappa^2}} \qquad \text{(Optical Transmission Spectrum)} \tag{1}$$

$$\mathcal{S}_{PP}[\Omega] = \mathcal{S}_{PP}^{\min} + \frac{\left(\mathcal{S}_{PP}^{\max} - \mathcal{S}_{PP}^{\min}\right)\left(\Gamma_m\Omega_m\right)^2}{\left(\Omega^2 - \Omega_m^2\right)^2 + \left(\Gamma_m\Omega\right)^2} \qquad \text{(Power Spectral Density)} \tag{2}$$

---

* ccr@ifi.unicamp.br
† gsw@unicamp.br

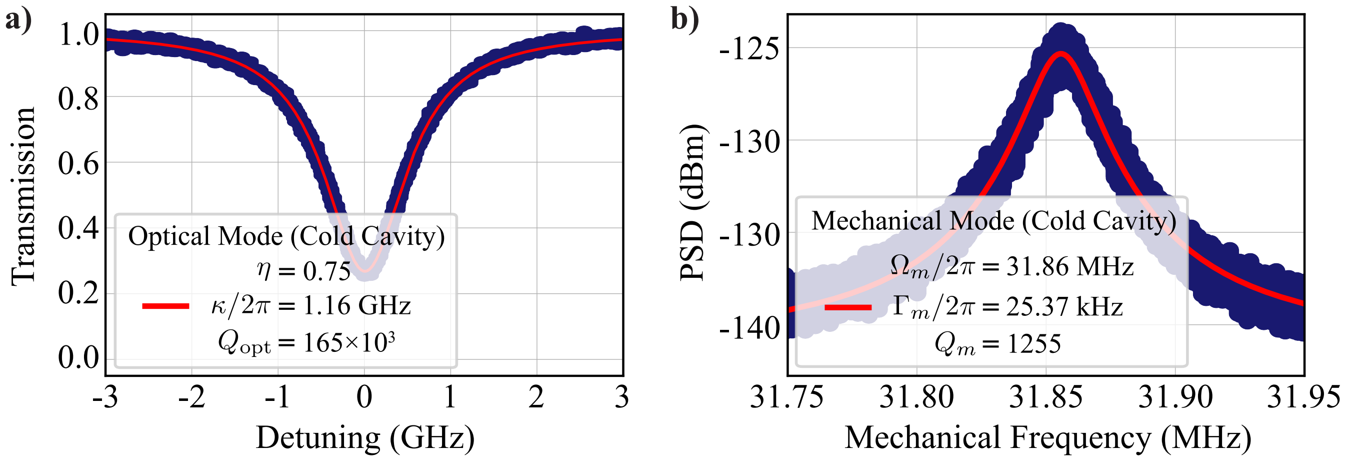

Supplementary Figure 2. **a)** Experimental optical transmission spectrum of the cavity; **b)** Experimental power spectrum density (PSD). The best fits of both curves are shown in red.

The value measured for the vacuum optomechanical coupling rate was $g_0/2\pi = 16.2$ kHz, where we have followed the M. L. Gorodetksy et al. article [3]. The function s_{in}^2 can be interpreted as the power reaching the cavity, i.e., $s_{\text{in}}^2 = P_{\text{in}}$, which is also valid for the output field $s_{\text{out}}^2 = P_{\text{out}}$. The power spectral density $\mathcal{S}_{PP}$ (or just PSD) shown in Equation (2) is in dBm units. The parameter $\eta = \kappa_e/\kappa$ is the coupling between the optical fiber taper and the cavity. For now on we are omitting the sub-index of $s_{\text{in}}(t) \to s(t)$, because we are not using s_{out} in any future calculations, so there will be no ambiguity in just writing $s(t)$ for the input field.

Supplementary Note 2.

Fractional Synchronization. As mentioned in the article we also observed several fractional order synchronizations, i.e., $\rho = p/q$ not an integer. We have shown in the main article, however, only the threshold to observe the tip of the Arnold tongues; here, we present in Supplementary Figure 3 the whole experimental map obtained.

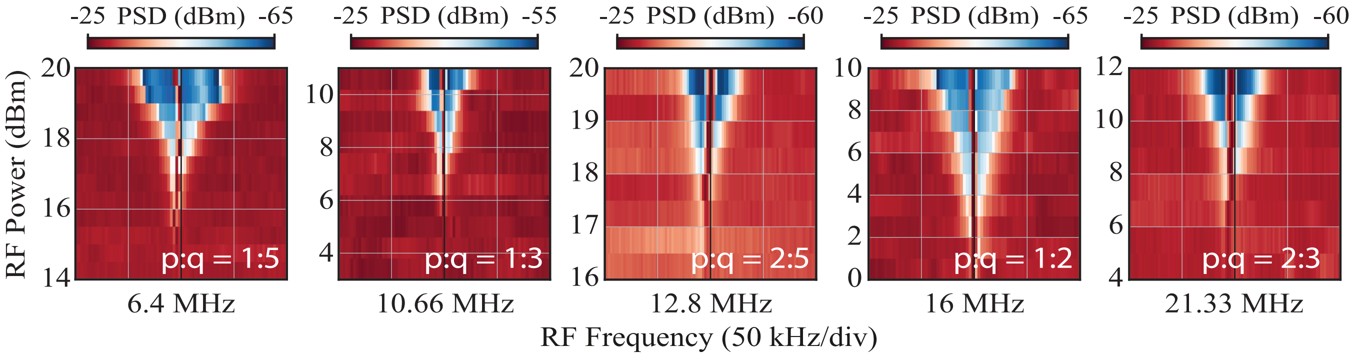

Supplementary Figure 3. Experimental fractional order Arnold tongues maps. The $p : q$ order is shown in white inside each map, which are arranged from lower frequency to higher frequencies from the left to the right.

These maps, however, requires a really strong modulation depth ε, impossible to study them in the weak perturbation regime of our semi-analytical model, as we are drastically changing the dynamic of the system. The importance of these data is to prove the existence of this kind of injection locking in optomechanics and to motivate the study of such phenomenon in future works, maybe the possibility of achieving such regimes using weak perturbations with different experimental parameters or even in some other cavity design to enhance these effects.

Supplementary Note 3.

Phase Noise Analysis and Routes to Frequency Division Optimization. In the main text we have shown only the phase noise measurements for the 4 : 1 injection, here we will show the PN density spectra for the high-harmonics, 2 : 1 to 4 : 1, as a function of the RF power, as shown in Supplementary Figure 4. For the smallest modulation depth (-19 dBm $\approx$ 1.5%) the 2 : 1 PN is flat around -70 dBc/Hz, in contrast with the 3 : 1 and 4 : 1 cases. For small modulations, both the 3 : 1 and 4 : 1 PN spectra appear to be transitioning from the OMO free-running spectrum to the injection-locked regime characterized by the flat plateau. This is expected as the farther we are from Ω_0 because smaller the interaction, in such a way that higher modulation depths are needed to achieve the same low PN levels.

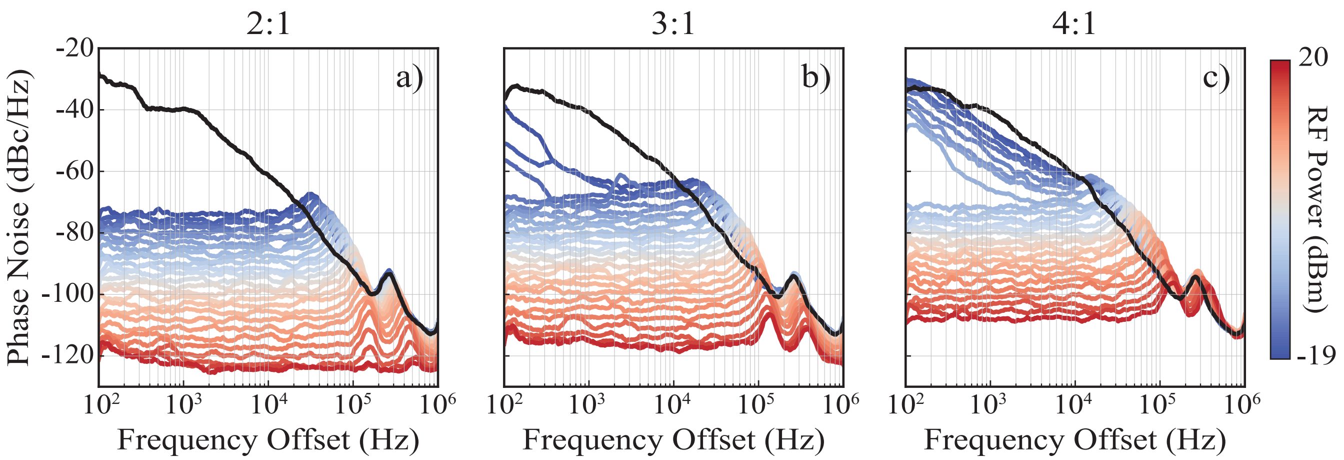

Supplementary Figure 4. Phase noise spectral densities for the cases 2 : 1, at **a)**, 3 : 1, at **b)**, and 4 : 1, **c)**, for various modulation depth. The black curve is the OMO free-running PN for reference.

A compact way to visualize the evolution of the phase noise as a function of the RF power is averaging the phase noise around low frequencies, as done in Supplementary Figure 5.

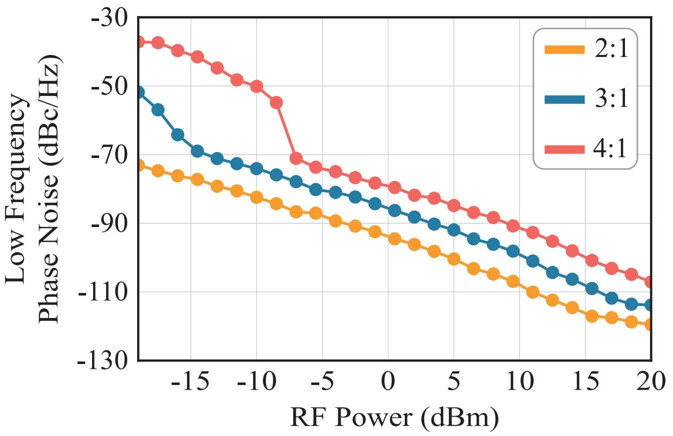

Supplementary Figure 5. Average phase noise around 100 Hz and 1 kHz for high harmonic injections as function of the RF power.

We address possible paths to optimize the injection locked phase noise. The model used for superharmonic injection phase noise is

$$\mathcal{L}_{\text{out}}(\Omega) = \frac{(\Delta\Omega_n/n)^2\mathcal{L}_{\text{inj}}(\Omega)\cos^2\theta + \Omega^2\mathcal{L}_{\text{free}}(\Omega)}{\Delta\Omega_n^2\cos^2\theta + \Omega^2}, \tag{3}$$

which is a weighted average of $\mathcal{L}_{\rm inj}(\Omega)$ and $\mathcal{L}_{\rm free}(\Omega)$. If any one of these is much smaller than the other, i.e., $\mathcal{L}_{\rm inj}(\Omega) \ll \mathcal{L}_{\rm free}(\Omega)$, we *cannot* expect $\mathcal{L}_{\rm out}(\Omega) \approx \mathcal{L}_{\rm inj}(\Omega)$, which would be desirable to a frequency divider. That being said, we must find ways to improve $\mathcal{L}_{\rm free}(\Omega)$ such that $\mathcal{L}_{\rm free}(\Omega)$ approaches $\mathcal{L}_{\rm inj}(\Omega)$. According to [2] and [5], an optomechanical cavity in the unresolved sideband regime is dominated by thermomechanical noise and its phase noise spectral density is given by

$$\mathcal{L}_{\rm free}(\Omega) = \left(\frac{2\Gamma_m}{n_x}\right)\left(\bar{n}_{\rm th} + \frac{1}{2}\right)\left(\frac{1}{\Omega^2} + \frac{\nu'^2_{\rm om}}{\Omega^2}\frac{1}{\gamma'^2_{\rm om} + \Omega^2} + \frac{\eta_I^2}{\gamma'^2_{\rm om} + \Omega^2}\right), \tag{4}$$

in which three "general rules" towards phase noise reduction are noticed: reducing Γ_m (mechanical oscillator's linewidth); reducing $\bar{n}_{\rm th}$ (thermal phonon number); and increasing n_x (coherent phonon number).

Quality factors up to $Q_m \approx 10000$, 10 times larger than in our experiment, are reported in silicon nitride double disks [9]. Such large mechanical quality factors were obtained just by manipulating the thickness of the nitride film. Also, increasing the optical pump power P_0 should readily increase the coherent phonon occupation n_x. To obtain an estimate on a feasible amplitude enhancement, we simulate the oscillation amplitude x (in units of its zero point fluctuation, $x_{\rm zpf}$) as a function of the optical detuning Δ_x, where the subindex indicates that we already counted the static optomechanical shift x_0, i.e., $\Delta_x = \omega_l - \omega_0 - Gx_0$. Our simulations were performed for a set of optical powers, as shown in Supplementary Figure 6, to find out the precise scaling of n_x with P_0. The numerical simulation details and the parameters used are covered in the next section.

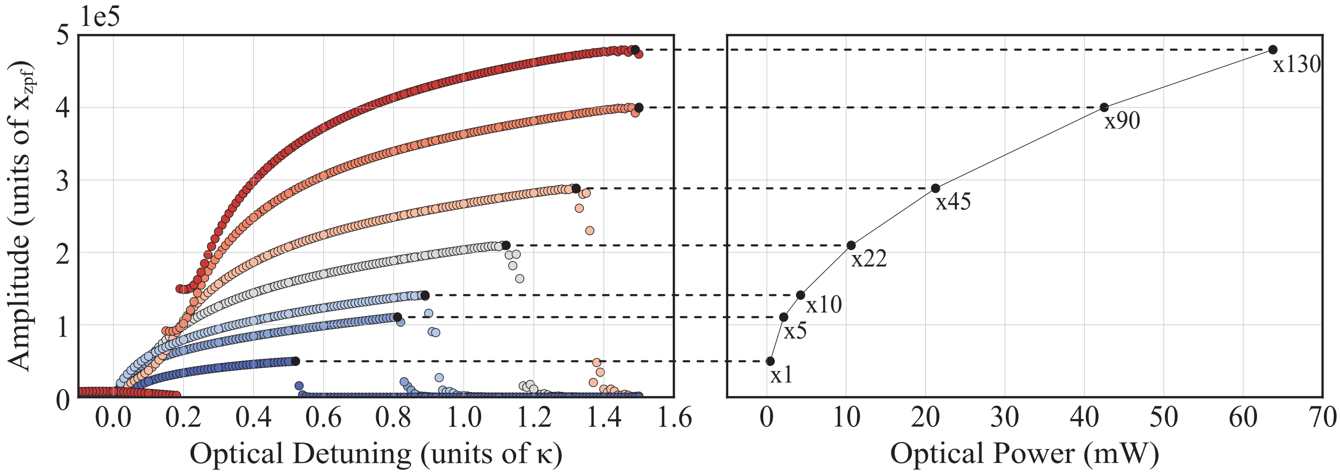

Supplementary Figure 6. **a)** Mechanical oscillator's amplitude, in units of $x_{\rm zpf}$, as a function of the optical detuning Δ_x, in units of κ; **b)** Maximum oscillator's amplitude for a given optical power P_0, in mW. The floating numbers indicates how many times we must multiply the actual pump power used in the experiment to reach that level. The $\times1$ case is the actual numerical simulation using the data obtained in the laboratory, which is $P_0 = 480\mu$W.

The terms $\nu'_{\rm om}$, $\gamma'_{\rm om}$ and η_I are the frequency shift due to amplitude change at the limit cycle, the damping rate of the amplitude fluctuation and transfers from displacement amplitude noise to photon number phase noise, respectively [2]. As Equation (4) show us, all these terms also contribute to the phase noise level, however, as we are using an optomechanical cavity in an unresolved sideband regime, we are neglecting their contributions because the term $1/\Omega^2$ dominates. Therefore, combining higher quality factors with larger oscillation amplitudes, we could achieve a net 30 dBc/Hz improvement, paving the way towards future improvements in optomechanical frequency dividers.

Supplementary Note 4.

Numerical Simulation. The numerical simulations of this section are not straightforward to perform, and it is worth discussing how they were carried out carefully. One issue faced when solving the following coupled nonlinear ODE

$$\dot{a} = i\Delta(t)a - \frac{\kappa}{2}a - iGxa + \sqrt{\kappa_e}s_0\sqrt{1+\varepsilon(t)\sin\Theta_d(t)} \qquad \text{and} \qquad \ddot{x} + \Gamma_m\dot{x} + \Omega_m^2 x = -\frac{\hbar G}{m_{\text{eff}}}|a|^2 \tag{5}$$

is the stiff nature of the system, characterized by the need of very small discretization steps despite the relative smoothness of the solutions. To tackle this system, we used well known numerical packages DifferentialEquations.jl, FFTW.jl, Sundials.jl and DSP.jl available in Julia language which implements robust methods for such systems. The simulation was done as follows: we first set an optical detuning function $\Delta(t)$ to sweep linearly from Δ_i to Δ_f, where the sub-indexes i and f means initial and final, respectively. We have chosen $\Delta_i > 0$ because we want to access the blue side of the optical mode, where the self-sustained dynamic is naturally accessible. After reaching Δ_f, we wait a few cycles of the mechanical oscillator to make sure the system is in a stationary regime and then turned on the modulation depth $\varepsilon(t)$, in which we modeled as a Heaviside step function. With the modulation depth online we, once again, waited a few microseconds to stabilize the energy inside the cavity, and then finally turned on the RF frequency sweep. In the laboratory our RF frequency sweep was linear between Ω_d^i and Ω_d^f with constant velocity $d\Omega_d/dt = \dot{\Omega}_d$, so we modeled $\Theta_d(t)$ as a parabola, i.e., $d\Theta_d(t)/dt = \Omega_d(t) = \Omega_d^i + \dot{\Omega}_d t$. The value chosen for $\dot{\Omega}_d$ need to be small to guarantee adiabaticity, which clearly is the case in the laboratory. A good threshold for adiabaticity is to sweep the RF tone over the mechanical resonance (of linewidth Γ_m) within the mechanical lifetime, $\tau_m \approx 2\pi/\Gamma_m$, i.e., $\dot{\Omega}_d \approx \Gamma_m/\tau_m \approx \Gamma_m^2/2\pi$. For our purposes, a RF frequency sweep velocity of $\dot{\Omega}_d \approx 0.1\Gamma_m^2$ was enough to ensure adiabaticity. A summary of all said is shown in Supplementary Figure 7, highlighting the main aspects of the dynamic.

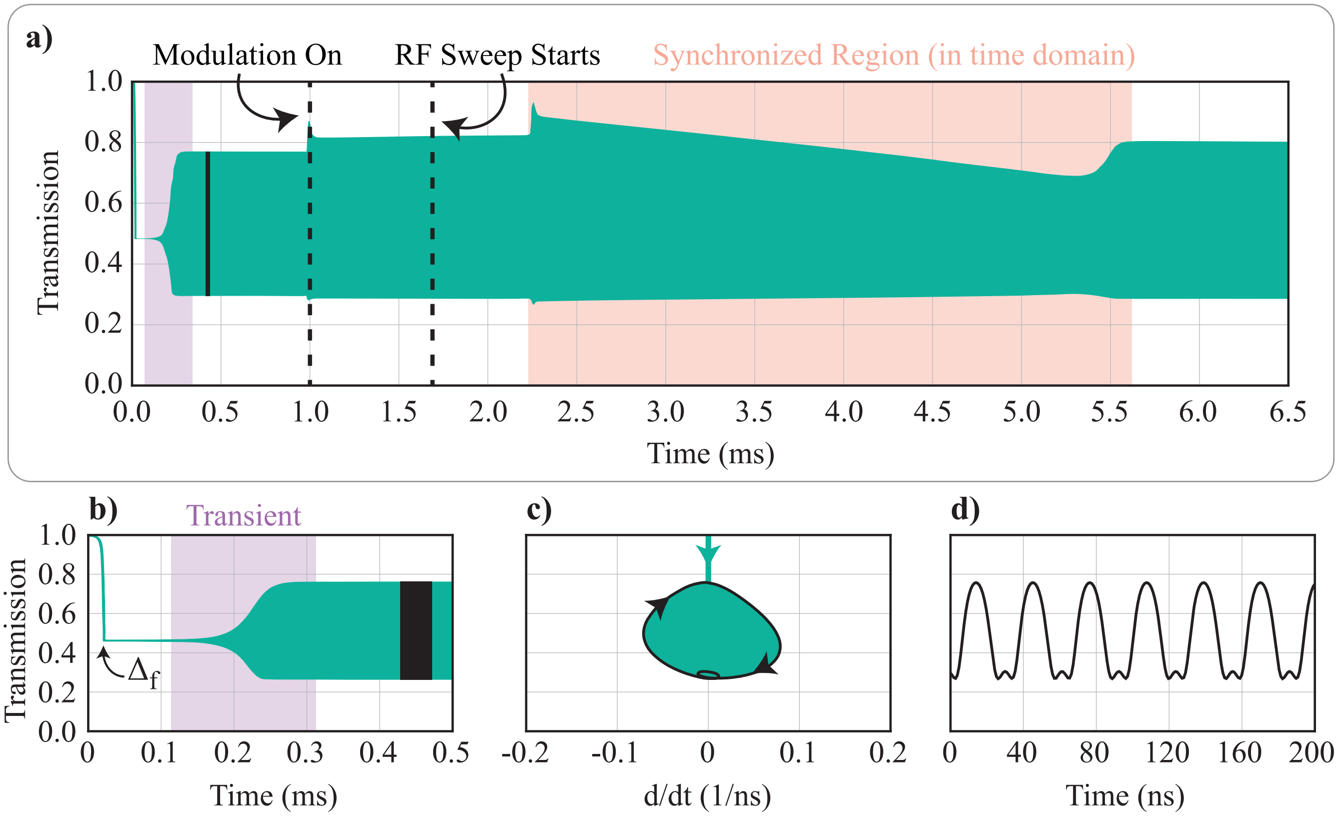

Supplementary Figure 7. **a)** Complete time domain simulation, showing important aspects of the synchronization. The purple region is the transient region which the mechanical oscillator gains amplitude. The two vertical dashed black lines shows where exactly we turned on the modulation ε and the RF sweep $\dot{\Omega}_d$. The pink region is where injection locking is happening; **b)** Transient region of Supplementary Figure 7(a) showing the regime from non-oscillating cavity to self-sustained oscillation; **c)** Phase space of Supplementary Figure 7(b); **d)** Temporal trace of the black part of Supplementary Figure 7(b).

These are the raw data that we obtain from the simulation. To obtain from these data the Arnold tongues we can take the length of the synchronized region of Supplementary Figure 7(a) - the pink region of the plot - for each modulation depth ε. However, we must clarify how we find this pink region, i.e., the specific point where we say that synchronization occurs is a bit blur in the time domain, and that's why we construct a spectrogram, which is the Fourier transform of our signal in function of time, as shown in Supplementary Figure 8(a). However, instead of plotting the spectrogram as a function of time, as we know the value of the driving RF frequency Ω_d for each time t, we can plot the spectrogram already in function of the RF frequency, and that is what was done in Supplementary Figure 8(a). One way to obtain the synchronized region is to take the horizontal slice of this spectrogram just above the mechanical oscillation frequency $\Omega_0/2\pi$, which is the horizontal dashed red line, and its plot is shown in Supplementary Figure 8(b). A second way, which is more well known in the literature, is to plot the difference between the driving frequency and oscillator's frequency ($\Omega_d - \Omega_0$) as a function of the drive frequency itself (or, in our case, the driving frequency minus a constant natural mechanical frequency Ω_m), as shown in Supplementary Figure 8(c).

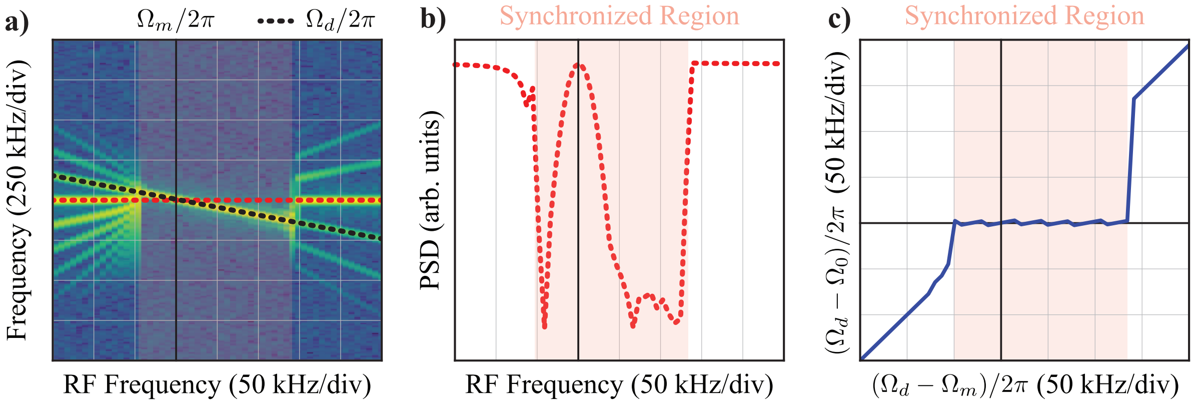

Supplementary Figure 8. **a)** Spectrogram of the transmission signal (Supplementary Figure 7(a)) after the RF sweep is turned on. The dashed black line is the value of the drive frequency. The vertical bold black line is the value of the mechanical mode frequency; **b)** Horizontal dashed red slice of the spectrogram shown in **a)**; **c)** Typical plot of synchronization systems showing the mismatch from driving frequency and bare oscillation frequency, making clear where these became the same, defining a synchronized state. We have used $\varepsilon = 2\%$ for these simulations.

The Arnold tongues constructed using the explanation above are shown in Supplementary Figure 9, which was already presented at the article as Fig. 3(a). As we can see, the simulation shows bigger synchronized region for the case $p : q = 2 : 1$ than $1 : 1$, and also a pretty wide $4 : 1$ AT, but a small $3 : 1$, the same trend of the experimental data. Supplementary Table 1 shows the parameters used in the simulations done and Supplementary Figure 10 shows the conversion from RF power, in dBm, to modulation depth ε, in %, which is based in experimental data. The actual formula for the RF power P_{RF} is shown inside the plot.

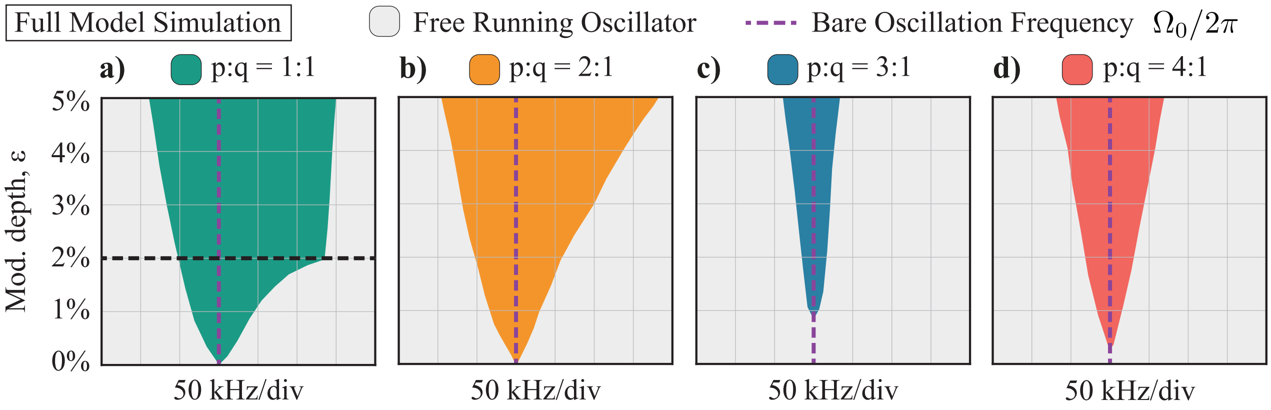

Supplementary Figure 9. Simulated Arnold tongues using injection frequency $\Omega_d = p\Omega_0/q$ for the cases $p = \{1, 2, 3, 4\}$ and $q = 1$, in order, from **a)** to **d)**. To simulate these maps we used Equation (5), being these the ones that we will always call "full model". The dashed black line at **a)** is the region that we analyzed in Supplementary Figure 8(c).

Parameters	Values
P_0	425 μW
λ	1560nm
Δ_i	8κ
Δ_f	0.35κ
$d\Delta/dt$	$10^2\Gamma_m^2$
$d\Omega_d/dt$	$0.075\Gamma_m^2$
η	0.75
$\kappa/2\pi$	1.16 GHz
Q_{opt}	165000
$\Omega_m/2\pi$	31.86 MHz
$\Gamma_m/2\pi$	25.37 kHz
Q_m	1255
$g_0/2\pi$	16.2 kHz

Supplementary Table 1. Parameters values used in every simulations, unless explicitly mentioned the opposite.

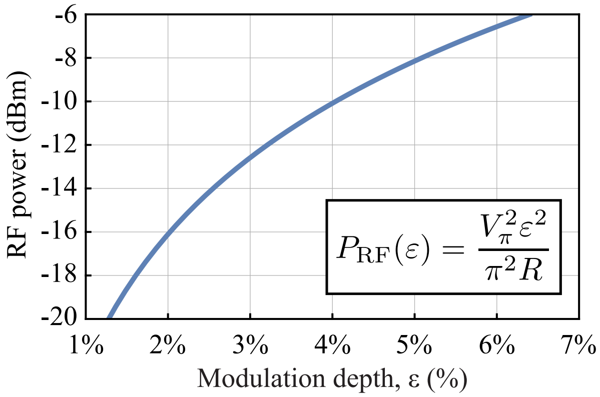

Supplementary Figure 10. Conversion from modulation depth, in percentage, to RF power, in dBm. The inset shows the actual formula, in S.I. units, of this graph. $V_\pi = 5.5$ V and $R = 50\ \Omega$.

Supplementary Note 5.

Semi-Analytical Model. The Hamiltonian of our system, regardless dissipative considerations, can be modeled by

$$H = \hbar\left(\omega_0 + Gx\right)a^\dagger a + \frac{p^2}{2m_{\text{eff}}} + \frac{m_{\text{eff}}\Omega_m x^2}{2} + i\hbar\sqrt{\kappa_e}s_0\left(e^{-i\omega_l t}a^\dagger - e^{i\omega_l t}a\right), \tag{6}$$

in which $\hbar$ is the reduced Planck's constant, ω_0 is the unperturbed angular frequency of the optical mode, $a^\dagger$ and a are the creation and annihilation operators for photons with energy $\hbar\omega_0$, respectively, G is the first order coefficient of the Taylor expansion of $\omega(x) = \omega_0 + Gx$ evaluated at mechanical equilibrium position (i.e., $G = (d\omega/dx)|_{x=0}$), p and x are the momentum and position operator of the mechanical oscillator, respectively, Ω_m is the unperturbed angular frequency of the mechanical mode, m_{eff} is the mechanical oscillator effective mass, i is the complex unity, κ_e is the external optical coupling rate, s_0^2 is the input power and ω_l is the optical pump angular frequency. As long as we are not interested in quantum phenomena we can study our dynamical system just looking to the average value of these operators, and we can also introduce the optical and mechanical loses κ and Γ_m directly in the equations of motion [1] as

$$\dot{a} = i\Delta a - \frac{\kappa}{2}a - iGxa + \sqrt{\kappa_e}s_0 \qquad \text{and} \qquad \ddot{x} + \Gamma_m\dot{x} + \Omega_m^2 x = -\frac{\hbar G}{m_{\text{eff}}}|a|^2, \tag{7}$$

where we already changed a to the slow rotating frame of reference $a \to ae^{-i\omega_l t}$ to let the equation autonomous. We define the bare optical detuning $\Delta = \omega_l - \omega_0$ as the difference between optical pump frequency and unperturbed optical mode frequency. To introduce the amplitude modulation used in the experiment we can simply multiply s_0 by a factor $\sqrt{1 + \varepsilon\sin\Theta_d(t)}$ in Equation (7), i.e., $s(t) = s_0\sqrt{1 + \varepsilon\sin\Theta_d(t)}$, in which ε is the modulation depth and is correlated with the RF power as shown in Supplementary Figure 10. The term $\Theta_d(t)$ is the phase of this modulation, in which most of the time will just be $\Omega_d t$. Simulating Equation (7) as it is shown require us to know G and m_{eff} but, because they are normalization dependent, we are going to avoid this using $g_0 = Gx_{\text{zpf}}$, the optomechanical single-photon coupling strength, and also the $x_{\text{zpf}} = \sqrt{\hbar/2m_{\text{eff}}\Omega_m}$, the zero points fluctuation amplitude of x. New normalizations will be used to study self-sustained oscillations, which are given by

$$x(t) = x_0 + \delta x(t) = \left(\frac{\kappa}{2g_0}\right)\sqrt{\frac{\hbar}{2m_{\text{eff}}\Omega_m}}\left(\widetilde{x}_0 + \widetilde{x}(t)\right) \qquad \text{and} \qquad t = \frac{\widetilde{t}}{\Omega_m}, \tag{8}$$

where all tilde variables are now adimensional. The terms x_0 and $\delta x(t)$ are the DC and AC components of $x(t)$, respectively, being $\widetilde{x}_0$ and $\widetilde{x}(t)$ their adimensional version. We can then rewrite Equation (7) as

$$\frac{da}{d\widetilde{t}} = -\frac{\kappa}{2\Omega_m}a + i\left(\frac{\Delta_x}{\Omega_m} - \frac{\kappa}{2\Omega_m}\widetilde{x}\right)a + \sqrt{\frac{\kappa_e}{\Omega_m}}\frac{s}{\sqrt{\Omega_m}} \qquad \text{and} \qquad \frac{d^2\widetilde{x}}{d\widetilde{t}^2} + \frac{1}{Q_m}\frac{d\widetilde{x}}{d\widetilde{t}} + \widetilde{x} = -\widetilde{x}_0 - \frac{\mathcal{C}_0}{Q_m}|a|^2, \tag{9}$$

where the new variables Δ_x, Q_m and $\mathcal{C}_0$ are the optical detuning with the static optomechanical shift correction, $\Delta_x = \omega_l - \omega_0 - Gx_0$, the mechanical oscillator quality factor, $Q_m = \Omega_m/\Gamma_m$, and the single-photon cooperativity $\mathcal{C}_0 = 4g_0^2/\Gamma_m\kappa$, respectively. The derivatives now will be all with respect to $\widetilde{t}$ unless explicitly said the opposite. The motivations to construct a semi-analytical model in this article are (i) to prove that each term F_n from the power expansion of the optical force is mainly responsible for the $p:q$ Arnold tongue width $\Delta\Omega(p,q)$, (ii) to obtain a semi-analytical formula for these $\Delta\Omega(p,q)$, (iii) to show that the influence of the optical detuning greatly change the synchronization region, (iv) to prove that the symmetry breaking term F_2, which is neglected in many articles, is actually crucial for the dynamic and (v) to explain/predict those sidebands around the synchronization region. We then start uncoupling Equation (9) using adiabatic considerations: our optomechanical cavity has mechanical linewidth Γ_m much smaller than the optical linewidth κ, as well the mechanical frequency Ω_m also much smaller than κ, a regime called unresolved sidebands. We can then assume that $a(\widetilde{t})$ is always in equilibrium with $\widetilde{x}(\widetilde{t}-\widetilde{\tau})$, where $\widetilde{\tau}$ is some adimensional time delay that we will deduce later in Equation (23), so we can write $a(\widetilde{t})$ as

$$a(\widetilde{t}) \approx \sqrt{\frac{\kappa_e}{\kappa}}\frac{s(\widetilde{t})}{\sqrt{\kappa}}\frac{2}{1-i\left[\frac{2\Delta_x}{\kappa}-\widetilde{x}(\widetilde{t}-\widetilde{\tau})\right]}, \tag{10}$$

and then we can analyze the whole system just looking to one equation

$$\ddot{\widetilde{x}}(\widetilde{t}) + \frac{\dot{\widetilde{x}}(\widetilde{t})}{Q_m} + \widetilde{x}(\widetilde{t}) = -\widetilde{x}_0 - \frac{\mathcal{C}_0}{Q_m}\left(\frac{2\kappa_e}{\kappa}\right)\left(\frac{2s_0^2}{\kappa}\right)\left(\frac{1+\varepsilon\sin\Theta_d(\widetilde{t})}{1+\left[\frac{2\Delta_x}{\kappa}-\widetilde{x}(\widetilde{t}-\widetilde{\tau})\right]^2}\right), \tag{11}$$

where we define $f(\widetilde{t})$ and f_0 as

$$f(\widetilde{t}) = f_0\left[1+\varepsilon\sin\Theta_d(\widetilde{t})\right] \quad \text{and} \quad f_0 = \frac{\mathcal{C}_0}{Q_m}\left(\frac{2\kappa_e}{\kappa}\right)\left(\frac{2s_0^2}{\kappa}\right), \tag{12}$$

which allows us to rewrite Equation (10) as

$$\ddot{\widetilde{x}}(\widetilde{t}) + \frac{\dot{\widetilde{x}}(\widetilde{t})}{Q_m} + \widetilde{x}(\widetilde{t}) = -\widetilde{x}_0 - \frac{f(\widetilde{t})}{1+\left[\frac{2\Delta_x}{\kappa}-\widetilde{x}(\widetilde{t}-\widetilde{\tau})\right]^2}, \tag{13}$$

However, Equation (13) is still very complicated because it is a non-autonomous delay differential equation, so we will expand the RHS in a power series of $\widetilde{x}(\widetilde{t}-\widetilde{\tau})$ as

$$\frac{1}{1+\left[\frac{2\Delta_x}{\kappa}-\widetilde{x}(\widetilde{t}-\widetilde{\tau})\right]^2} = F_0 + F_1\widetilde{x}(\widetilde{t}-\widetilde{\tau}) + F_2\widetilde{x}^2(\widetilde{t}-\widetilde{\tau}) + F_3\widetilde{x}^3(\widetilde{t}-\widetilde{\tau}) + ... \tag{14}$$

in which the actual form of these first coefficients (which were already shown in Fig. 1(b) of the article) are

$$F_0 = \frac{1}{1+\frac{4\Delta_x^2}{\kappa^2}} \quad , \quad F_1 = \frac{2\left(\frac{2\Delta_x}{\kappa}\right)}{\left(1+\frac{4\Delta_x^2}{\kappa^2}\right)^2} \quad , \quad F_2 = \frac{\left(\frac{12\Delta_x^2}{\kappa^2}-1\right)}{\left(1+\frac{4\Delta_x^2}{\kappa^2}\right)^3} \quad \text{and} \quad F_3 = \frac{4\left(\frac{2\Delta_x}{\kappa}\right)\left(\frac{4\Delta_x^2}{\kappa^2}-1\right)}{\left(1+\frac{4\Delta_x^2}{\kappa^2}\right)^4}, \tag{15}$$

which shows that large normalized detuning ($\Delta_x/\kappa \gg 1$) leads to negligible values, as each F_{n+1} term decreases faster than F_n as a function of Δ_x/κ, i.e.,

$$\frac{F_n}{F_{n+1}} \sim \left(\frac{\Delta_x}{\kappa}\right). \tag{16}$$

The value of each F_n in our experiment was found to be $F_0 = 0.6711$, $F_1 = 0.6306$, $F_2 = 0.1421$ and $F_3 = -0.2897$. Substituting Equation (14) in Equation (13) reveals the nonlinear nature of the optical feedback into the mechanical oscillator,

$$\ddot{\widetilde{x}}(\widetilde{t}) + \frac{\dot{\widetilde{x}}(\widetilde{t})}{Q_m} + \widetilde{x}(\widetilde{t}) = -\widetilde{x}_0 - f(\widetilde{t})\left[F_0 + F_1\widetilde{x}(\widetilde{t}-\widetilde{\tau}) + F_2\widetilde{x}^2(\widetilde{t}-\widetilde{\tau}) + F_3\widetilde{x}^3(\widetilde{t}-\widetilde{\tau})\right]. \tag{17}$$

To remove the delay dependence we can expand $\widetilde{x}(\widetilde{t}-\widetilde{\tau})$ in powers of $\widetilde{\tau}$ as

$$\widetilde{x}^n(\widetilde{t}-\widetilde{\tau}) = \widetilde{x}^n(\widetilde{t}) - n\widetilde{\tau}\widetilde{x}^{n-1}(\widetilde{t})\dot{\widetilde{x}}(\widetilde{t}) + O(\widetilde{\tau}^2), \tag{18}$$

where we are neglecting $O(\widetilde{\tau}^2)$ since we have that $\widetilde{\tau}^2$ is of order $O(\Omega_m^2/\kappa^2)$, as we will verify soon. We can then group all these terms in an arrangement that highlights how far from the ideal harmonic oscillator this system is, as shown in Equation (19)

$$\ddot{\widetilde{x}} + \left[\frac{1}{Q_m} - \widetilde{\tau} f(\widetilde{t})\left(F_1 + 2F_2\widetilde{x} + 3F_3\widetilde{x}^2\right)\right]\dot{\widetilde{x}} + \left[1 + f(\widetilde{t})\left(F_1 + F_2\widetilde{x} + F_3\widetilde{x}^2\right)\right]\widetilde{x} = -f_1(\widetilde{t})F_0 - \left(f_0F_0 + \widetilde{x}_0\right), \tag{19}$$

where $f_1(\widetilde{t})$ is the AC component of $f(\widetilde{t})$, i.e., $f(\widetilde{t}) = f_0 + f_1(\widetilde{t})$ with $f_1(\widetilde{t}) = \varepsilon f_0 \sin\Theta_d(\widetilde{t})$. The last term of Equation (19) in parentheses must be zero because is the only DC component of the whole equation and, as one can verify, solving $f_0F_0 + \widetilde{x}_0 = 0$ returns the same static correction for the mechanical displacement $x(t)$ from the linearized optomechanics. Equation (19) is, finally, a well looking shape equation, very similar with Equation 8 from reference [7] where their ζ and f_e are related with our F_1 and F_0, besides we are not using temperature dynamics here. The generalization of Equation (19) until $O(\widetilde{\tau}^2)$ is given by

$$\ddot{\widetilde{x}} + \left[\frac{1}{Q_m} - \widetilde{\tau} f(\widetilde{t})\sum_{n=1}^{n_{\max}} nF_n\widetilde{x}^{n-1}\right]\dot{\widetilde{x}} + \left[1 + f(\widetilde{t})\sum_{n=1}^{n_{\max}} F_n\widetilde{x}^{n-1}\right]\widetilde{x} = -f_1(\widetilde{t})F_0, \tag{20}$$

but we are not going to analyze this system, we will stick with the case $n_{\max} = 3$. We can do a final approximation which is to neglect terms of order $O(\widetilde{\tau}\varepsilon)$ as far ε is kept small, knowing a priori that $\widetilde{\tau}$ is already small. We then have Equation (21)

$$\ddot{\widetilde{x}} + \left[\frac{1}{Q_m} - \widetilde{\tau} f_0\left(F_1 + 2F_2\widetilde{x} + 3F_3\widetilde{x}^2\right)\right]\dot{\widetilde{x}} + \left[1 + (f_0 + f_1)\left(F_1 + F_2\widetilde{x} + F_3\widetilde{x}^2\right)\right]\widetilde{x} = -f_1F_0 \tag{21}$$

and finally it is an ODE that all the terms proportional to $\widetilde{x}$ have a parametric excitation f_1, but terms proportional to $\dot{\widetilde{x}}$ do not. The only formula that is missing is $\widetilde{\tau} = \widetilde{\tau}(\Delta_x)$ for us to start studying Equation (21). To find this missing expression note (i) that the term f_0F_1 acts like a constant shift in the frequency, so we can associate with it the optical spring effect and (ii) that the term $-\widetilde{\tau} f_0F_1$ acts like a constant change in the mechanical linewidth, so we can associate it with the optical cooling/heating. Doing that interconnection with the linearized optomechanical equations [1] we can identify an analytic expression for $\widetilde{\tau}$ because from our model we have that $\delta\Gamma_m^{\text{linear}} = -\widetilde{\tau} f_0F_1$ and, from the linearized optomechanical equations,

$$\delta\Gamma_m^{\text{linear}} = \frac{\mathcal{C}_0}{Q_m}\left(\frac{2\kappa_e}{\kappa}\right)\left(\frac{2s_0^2}{\kappa}\right)\left(\frac{1}{1+\left(\frac{2\Delta_x}{\kappa}\right)^2}\right)\left(\frac{1}{1+\left(\frac{2\Delta_x}{\kappa}+\frac{2\Omega_m}{\kappa}\right)^2} - \frac{1}{1+\left(\frac{2\Delta_x}{\kappa}-\frac{2\Omega_m}{\kappa}\right)^2}\right), \tag{22}$$

remembering that $\delta\Gamma_m^{\text{linear}}$ is in units of Ω_m. Solving then for $\widetilde{\tau}$

$$\widetilde{\tau} = \frac{1}{2\left(\frac{2\Delta_x}{\kappa}\right)}\left[\frac{1+\left(\frac{2\Delta_x}{\kappa}\right)^2}{1+\left(\frac{2\Delta_x}{\kappa}-\frac{2\Omega_m}{\kappa}\right)^2} - \frac{1+\left(\frac{2\Delta_x}{\kappa}\right)^2}{1+\left(\frac{2\Delta_x}{\kappa}+\frac{2\Omega_m}{\kappa}\right)^2}\right]. \tag{23}$$

We must emphasize here that our $\widetilde{\tau}$ should, rigorously, be rewritten as $\widetilde{\tau}_{\text{linear}}$, because that is just the first order correction of $\widetilde{\tau}$. Nevertheless, the function $\widetilde{\tau} = \widetilde{\tau}(\Delta_x)$ has every property that we expect: (i) is positive for every value of Δ_x, (ii) it is approximately Ω_m/κ and (iii) is also consistent with the fact that far from the resonance there is no mechanical response, i.e., $\widetilde{\tau}(|\Delta_x| \gg \kappa) = 0$, as shown in Supplementary Figure 11 using our experimental parameters and also for three other cases.

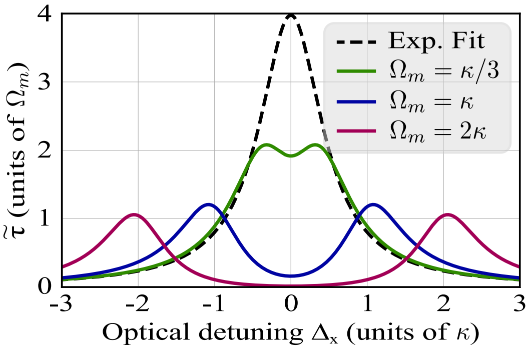

Supplementary Figure 11. Adimensional linear mechanical relaxation time $\widetilde{\tau}$ (in units of Ω_m/κ) as function of the optical detuning Δ_x (in units of κ).

The Arnold tongues simulations using this semi-analytical model from Equation (21) are shown in Supplementary Figure 12, and the comparison between this and Supplementary Figure 9 is striking. Before finishing this section we want to prove that the connection between F_n's and the entire AT width $\Delta\Omega(n+1,1)$ is really strong and, for that, we simulated again Equation (21) but here we want to show not the AT map, but some particular spectrograms while we considered only one F_n each time we simulate the system, as shown in Supplementary Figure 13.

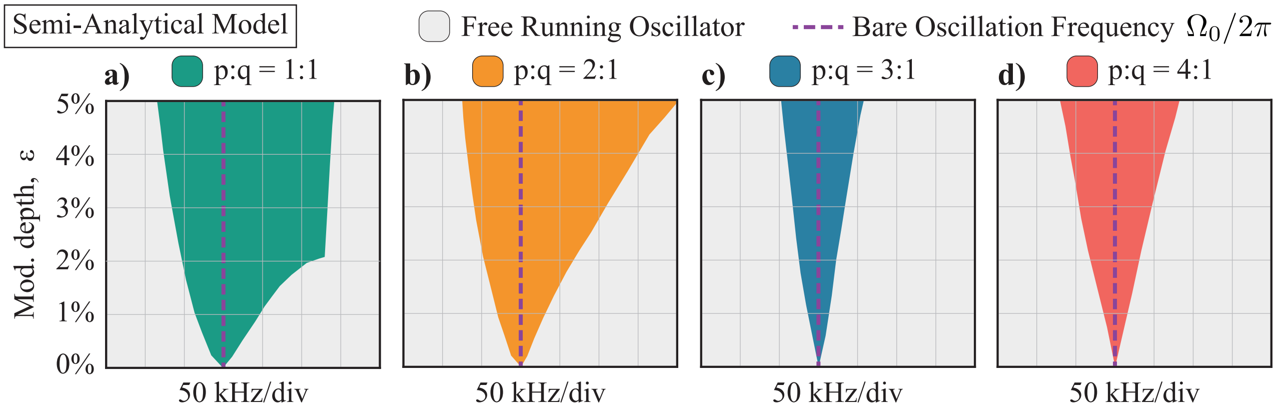

Supplementary Figure 12. Simulated Arnold tongues using injection frequency $\Omega_d = p\Omega_0/q$ for the cases $p = \{1, 2, 3, 4\}$ and $q = 1$, in order, from **a)** to **d)**. To simulate these maps we used Equation (21).

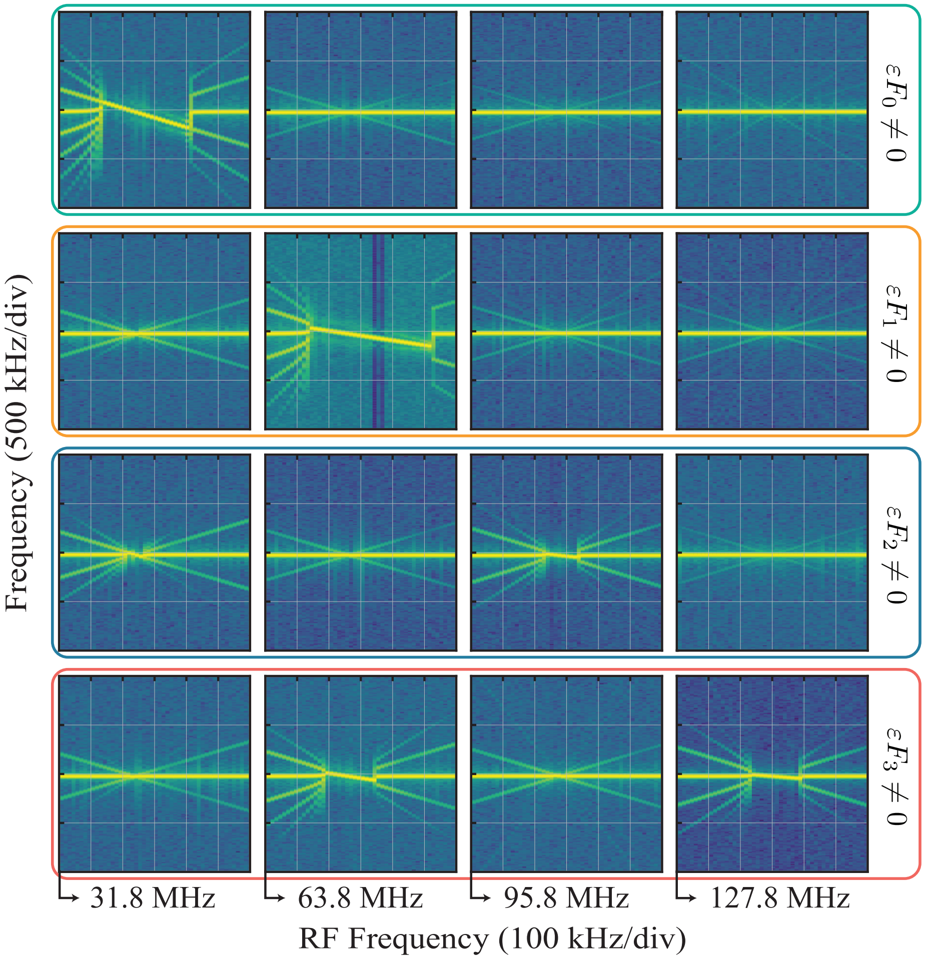

Supplementary Figure 13. These simulations were done considering only one εF_n term in Equation (21), e.g., the first row of this image-matrix-like consist of considering $\varepsilon F_1 = \varepsilon F_2 = \varepsilon F_3 = 0$ while $\varepsilon F_0 \neq 0$ and doing the injection locking for all $p = 1 - 4$. The same logic is valid for the others rows, as indicated in the right side of the figure. In these simulations we used $\varepsilon = 5\%$.

And as we can see, simulating Equation (21) proves that the major dependence of each AT is indeed the parametric term εF_n, as each one of these terms alone almost reproduces the whole dynamic of the system in a specific region. This hierarchical dependence is explicitly calculated, as shown in Equation (48).

Supplementary Note 6.

The averaging method of Krylov-Bogoliubov-Mitropolsky (KBM). While the numerical simulation does predict many features and give us many insights about the observed data, it does not provide a direct prediction of the synchronization behavior. To pursue further analytical insight, we resort to the KBM method to derive amplitude and phase equations describing the coupling optomechanical oscillator enslaved by the driving modulated RF signal. We start here introducing new adimensional time T and displacement y given by

$$\frac{\widetilde{x}}{y} = L_{\widetilde{x}} = -\frac{F_2}{3F_3} + \sqrt{\left(\frac{F_2}{3F_3}\right)^2 + \left(\frac{1 - f_0 F_1 Q_m \widetilde{\tau}}{3 f_0 F_3 Q_m \widetilde{\tau}}\right)} \qquad \text{and} \qquad \frac{\widetilde{t}}{T} = L_{\widetilde{t}} = \frac{1}{\sqrt{1 + f_0 F_1}}. \tag{24}$$

At first glance, it seems like an awkward choice of normalization, but as we discuss below, they have a clear physical interpretation. Equation (24) is the positive root of the coefficient of $\dot{\widetilde{x}}(\widetilde{t})$ in Equation (21), which makes the amplitude of y near the value of the limit circle of a van-der Pol oscillator $\approx O(1)$, i.e., we are renormalizing $\widetilde{x}$ by the positive solution of

$$\frac{1}{Q_m} - \widetilde{\tau} f_0 \left(F_1 + 2F_2 \widetilde{x} + 3F_3 \widetilde{x}^2\right) = 0. \tag{25}$$

The choice of the new time scale makes the oscillation frequency of the oscillator, already accounted by the optical spring effect, about $\approx O(1)$. After these normalizations we have that Equation (21) becomes

$$\frac{d^2 y}{dT^2} - \mu(1-y)(1+\sigma y)\frac{dy}{dT} + \left[1 + \varepsilon\alpha \sin(\omega T)\right] y + \\ + \beta \left[1 + \varepsilon \sin(\omega T)\right] y^2 + \gamma \left[1 + \varepsilon \sin(\omega T)\right] y^3 = F\varepsilon \sin(\omega T), \tag{26}$$

with new adimensional parameters defined as

$$\omega = \frac{\Omega_d}{\Omega_m} L_{\widetilde{t}}, \qquad \mu = \left(\widetilde{\tau} f_0 F_1 Q_m - 1\right) L_{\widetilde{t}}, \qquad \sigma = 1 + \frac{2\widetilde{\tau} f_0 F_2 Q_m}{\widetilde{\tau} f_0 F_1 Q_m - 1} L_{\widetilde{x}}, \tag{27}$$

$$\alpha = f_0 F_1 L_{\widetilde{t}}^2, \qquad \beta = f_0 F_2 L_{\widetilde{x}} L_{\widetilde{t}}^2, \qquad \gamma = f_0 F_3 L_{\widetilde{x}}^2 L_{\widetilde{t}}^2, \qquad \text{and} \qquad F = -f_0 F_0 L_{\widetilde{t}}^2 / L_{\widetilde{x}}.$$

It is evident that every parametric term α, β, γ and F is proportional to f_0, regardless of the convoluted terms $L_{\widetilde{t}}$ and $L_{\widetilde{x}}$, meaning that higher optical pump intensity enhance these terms. Also, each of these terms are proportional to one F_n, making clear distinction where each nonlinearity really is. Such model returns us the same used by Shreyas Y. Shah at [6] if $\mu = \gamma = F = 0$ and also neglecting the autonomous quadratic term βy^2 (which is the term that comes from a odd power potential, making the problem parity asymmetric), then

$$\frac{d^2 y}{dT^2} + \left[1 + \alpha\varepsilon \sin(\omega T)\right] y + \beta\varepsilon \sin(\omega T) y^2 = 0, \tag{28}$$

but to leave in the exact shape of the one used there we should change the independent variable $\omega T \to U + \frac{\pi}{2}$ and then

$$\frac{d^2 y}{dU^2} + \left[\frac{1}{\omega^2} + \frac{\alpha}{\omega^2}\varepsilon \cos(U)\right] y + \frac{\beta}{\omega^2}\varepsilon \cos(U) y^2 = 0, \tag{29}$$

from which we interpret the parameters as

$$\delta^{\text{Shah}} = \frac{1}{\omega^2} \quad , \quad D_1^{\text{Shah}} = \frac{\alpha}{\omega^2} \quad , \quad D_2^{\text{Shah}} = \frac{\beta}{\omega^2} \quad , \quad \gamma^{\text{Shah}} = \varepsilon. \tag{30}$$

However, we will not use the multi-scale method to study synchronization neither to find bifurcations, our analysis will be based in the KBM method of averaging. The value of the parameters obtained from simulations are $\mu = 9.813 \times 10^{-4}$, $\sigma = 1.665 \times 10^0$, $\alpha = 2.383 \times 10^{-2}$, $\beta = 4.396 \times 10^{-3}$, $\gamma = -7.340 \times 10^{-3}$ and $F = -3.098 \times 10^{-2}$.

We begin our KBM analyzes from Equation (26), which is a nonlinear oscillators of the form

$$\frac{d^2 y}{dT^2} + y = K\left(T, y, \frac{dy}{dT}\right), \tag{31}$$

where K is small compared to y. If $K(T, y, \frac{dy}{dT}) = 0$, we would have the ideal harmonic oscillator with solution $y = A \sin(T + \Phi)$ for any choice of constants A and Φ. If we now try solving Equation (31) with slowly varying amplitude and phase $(A(T), \Phi(T))$ as ansatz, i.e.,

$$y = A(T) \sin[T + \Phi(T)] \qquad \text{and} \qquad \frac{dy}{dT} = A(T) \cos[T + \Phi(T)], \tag{32}$$

we can show [4] that this system has general solution given by Equation (33)

$$\begin{cases} \frac{dA}{dT} = \cos(\phi) K(T, A \sin\phi, A \cos\phi) \\ \\ \phi(T) = T + \Phi(T) \\ \\ \frac{d\Phi}{dT} = -\frac{\sin(\phi)}{A} K(T, A \sin\phi, A \cos\phi) \end{cases} \tag{33}$$

The KBM method take its place here, where we average these equations over one period, however, the integral in T is replaced over a integral in ϕ considering that $d\phi \approx dT$, which is correct to zero order in $\Phi(T)$, so

$$\left\langle \frac{dA}{dT} \right\rangle_T \approx \left\langle \frac{dA}{dT} \right\rangle_\phi = \frac{1}{2\pi} \int_0^{2\pi} \cos(\phi) K(\phi - \Phi, A \sin\phi, A \cos\phi)\, d\phi \tag{34}$$

$$\left\langle \frac{d\Phi}{dT} \right\rangle_T \approx \left\langle \frac{d\Phi}{dT} \right\rangle_\phi = -\frac{1}{2\pi A} \int_0^{2\pi} \sin(\phi) K(\phi - \Phi, A \sin\phi, A \cos\phi)\, d\phi \tag{35}$$

and if our system were autonomous the integrals from Equation (34) and Equation (35) would be relatively easy to proceed, however, we have an external drive and this makes our system non-autonomous. To proceed the integral, we need to deal with Φ, which we will just let constant during integration, arguing that Φ is a slow varying function of T. The general form of $K\left(T, y, \frac{dy}{dT}\right)$ for our system can be splitted in two contribution: one autonomous and another one non-autonomous, i.e.,

$$K\left(T, y, \frac{dy}{dT}\right) = K_{\text{auto}}\left(y, \frac{dy}{dT}\right) + K_{\text{non-auto}}\left(T, y, \frac{dy}{dT}\right) \tag{36}$$

in which each part is written as

$$K_{\text{auto}}\left(y, \frac{dy}{dT}\right) = \mu (1 - y)(1 + \sigma y) \frac{dy}{dT} - \beta y^2 - \gamma y^3 \tag{37}$$

$$\text{and} \qquad K_{\text{non-auto}}\left(T, y, \frac{dy}{dT}\right) = \varepsilon \sin(\omega T) \left(F - \alpha y - \beta y^2 - \gamma y^3\right). \tag{38}$$

Substituting $K\left(T, y, \frac{dy}{dT}\right)$ into Equation (34) and Equation (35) and then performing the integration over one period of ϕ we obtain our amplitude and phase differential equations given by

$$\left\langle \frac{dA}{dT} \right\rangle_\phi = \frac{\mu A}{2}\left(1 - \frac{\sigma A^2}{4}\right) + \\ + \frac{\varepsilon \sin(\pi\omega)}{\pi} \left(\frac{\omega\left[F\left(\omega^2 - 9\right) + 2\beta A^2\right] \sin[(\pi - \Phi)\omega]}{\left(\omega^2 - 9\right)\left(\omega^2 - 1\right)} - \frac{A\left[\alpha\left(\omega^2 - 16\right) - 6\gamma A^2\right] \cos[(\pi - \Phi)\omega]}{\left(\omega^2 - 16\right)\left(\omega^2 - 4\right)} \right), \tag{39}$$

$$\left\langle \frac{d\Phi}{dT} \right\rangle_\phi = \frac{3\gamma A^2}{8} + \\ - \frac{\varepsilon \sin(\pi\omega)}{\pi} \left(\frac{\left[F\left(\omega^2 - 9\right) + 6\beta A^2\right] \cos[(\pi - \Phi)\omega]}{\left(\omega^2 - 9\right)\left(\omega^2 - 1\right) A} - \frac{2\left[\alpha\left(\omega^2 - 16\right) - 12 A^2 \gamma\right] \sin[(\pi - \Phi)\omega]}{\omega\left(\omega^2 - 16\right)\left(\omega^2 - 4\right)} \right). \tag{40}$$

This averaging technique is the essence of the KBM method to obtain amplitude and phase equations of nonlinear oscillators. Before we proceed, we can study Equation (39) and Equation (40) for the case $\varepsilon = 0$, which would give us exact solutions for both $A(T)$ and $\Phi(T)$ as

$$\begin{cases} A(T) = \frac{\pm 2}{\sqrt{\sigma + \left(\frac{4}{A_0} - \sigma\right) e^{-\mu T}}} \quad \Rightarrow \quad \lim_{T\to\infty} A(T) = A_\infty = \pm \frac{2}{\sqrt{\sigma}} \\ \\ \Phi(T) = \Phi_0 + \frac{3\gamma}{2\mu\sigma} \ln\left[1 + \frac{\sigma A_0^2}{4}\left(e^{\mu T} - 1\right)\right] \quad \Rightarrow \quad \lim_{T\to\infty} \Phi(T) = \Phi_\infty = \frac{3\gamma}{2\sigma} T \end{cases} \tag{41}$$

for constants A_0 and Φ_0. Here we conclude that even with zero modulation depth there exist a frequency shift contribution that comes from the Duffing term γ. The steady oscillation frequency Ω_0 is given by

$$\Omega_0(\varepsilon = 0) = \lim_{t\to\infty} \frac{d\phi}{dt} = \lim_{t\to\infty} \frac{d\phi}{dT}\frac{dT}{dt} = \frac{\Omega_m}{L_{\tilde{t}}} \frac{d}{dT}(T + \Phi_\infty) = \Omega_m \sqrt{1 + f_0 F_1}\left(1 + \frac{3\gamma}{2\sigma}\right) \tag{42}$$

which can be used to estimate the Duffing term from the measured oscillation frequency. Remembering the definitions of ω and ρ we can obtain a really nice relation between them as

$$\omega = \frac{\Omega_d}{\Omega_m \sqrt{1 + f_0 F_1}} \qquad \& \qquad \rho = \frac{p}{q} = \frac{\Omega_d}{\Omega_0} \qquad \Rightarrow \qquad \frac{\omega}{\rho} = 1 + \frac{3\gamma}{2\sigma} + O(\varepsilon), \tag{43}$$

and its clear now that if ρ is an integer, it does not mean that ω is an integer. In our case (and most of the cases) the Duffing correction in the frequency is really small, as a matter of fact we have

$$\frac{3\gamma}{2\sigma} \approx -6.6125 \times 10^{-3} = O(10^{-3}) \ll O(1) \tag{44}$$

so we will considerer $\omega \approx \rho$ for now on, because that is an excellent approach to obtain simple, but good, analytical results. The phase equation can be expanded in the vicinity of some integer $\rho = \{1, 2, 3, 4\}$ to give us insight into higher harmonic synchronization. We have then the following cases

$$\begin{cases} \lim_{\rho\to 1} \left\langle \frac{d\Phi}{dT} \right\rangle_\phi \approx \frac{3\gamma A^2}{8} - \varepsilon\left(\frac{F}{2A} - \frac{3\beta A}{8}\right)\cos(\Phi), \\ \\ \lim_{\rho\to 2} \left\langle \frac{d\Phi}{dT} \right\rangle_\phi \approx \frac{3\gamma A^2}{8} + \varepsilon\left(\frac{\alpha}{4} + \frac{\gamma A^2}{4}\right)\sin(2\Phi). \\ \\ \lim_{\rho\to 3} \left\langle \frac{d\Phi}{dT} \right\rangle_\phi \approx \frac{3\gamma A^2}{8} - \varepsilon\frac{\beta A}{8}\cos(3\Phi). \\ \\ \lim_{\rho\to 4} \left\langle \frac{d\Phi}{dT} \right\rangle_\phi \approx \frac{3\gamma A^2}{8} - \varepsilon\frac{\gamma A^2}{16}\sin(4\Phi). \end{cases} \tag{45}$$

When the system is locked to the driving signal, we know that the amplitude $A(T)$ of the oscillator is almost constant (this is the Kuramoto approximation) and that the phase $\phi(T) = T + \Phi(T)$ is a linear function of time T because, otherwise, the oscillation frequency Ω_0 would not be static, i.e., it would fluctuate around some mean frequency. In other words, we are imposing that the derivative of $\phi(T)$ to be constant during the locking, so we can say that $\Phi(T) = \delta_\rho T$ for some frequency mismatch δ_ρ of our bare oscillator, i.e.,

$$\phi(T) = (1 + \delta_\rho)T \quad \Rightarrow \quad \frac{d\phi}{dT} = 1 + \delta_\rho, \tag{46}$$

and then we can solve for $\varepsilon = \varepsilon(\delta_\rho)$ for various δ_ρ. The cases $\rho = 1$ and $\rho = 2$ are

$$\begin{cases} \delta_1 = \frac{3\gamma A^2}{8} - \varepsilon\left(\frac{F}{2A} - \frac{3\beta A}{8}\right)\cos(\delta_1 T), \\ \\ \delta_2 = \frac{3\gamma A^2}{8} + \varepsilon\left(\frac{\alpha}{4} + \frac{\gamma A^2}{4}\right)\sin(2\delta_2 T), \end{cases} \quad \Rightarrow \quad \begin{cases} \varepsilon(\delta_1) > \left|\left(\delta_1 - \frac{3\gamma A^2}{8}\right) / \left(\frac{F}{2A} - \frac{3\beta A}{8}\right)\right|, \\ \\ \varepsilon(\delta_2) > \left|\left(\delta_2 - \frac{3\gamma A^2}{8}\right) / \left(\frac{\alpha}{4} + \frac{\gamma A^2}{4}\right)\right|, \end{cases} \tag{47}$$

which defines a region in a $\varepsilon - \delta_\rho$ space which is, as we would guess, the Arnold tongues. The AT maps using this approach are shown in Supplementary Figure 14 for three different oscillation amplitudes $A = \{2, 3, 4\}$, which has the same effects of ones obtained from the experiment even after lots of approximations.

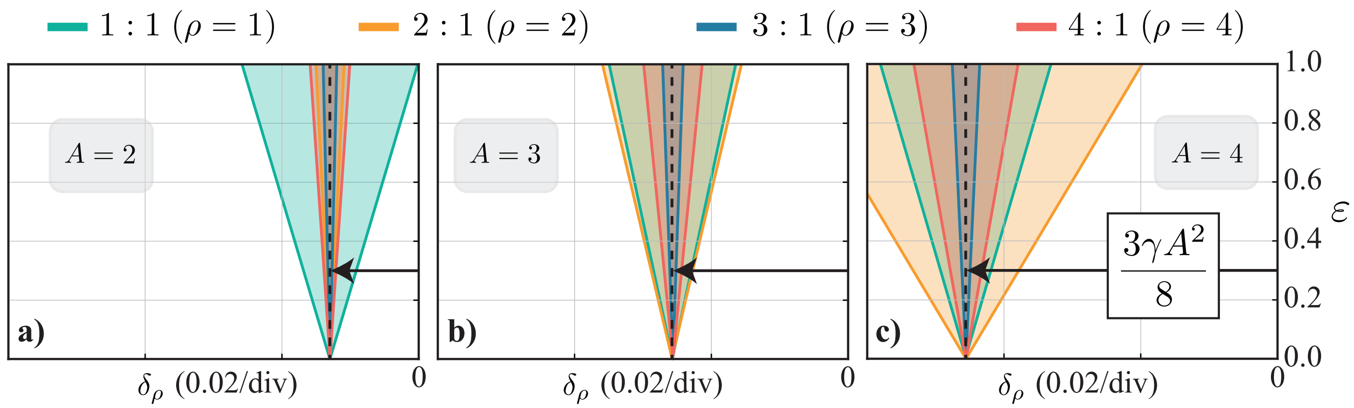

Supplementary Figure 14. Simulated Arnold tongues using Equation (45) for three different oscillations amplitude. **a)** $A = 2$; **b)** $A = 3$; **c)** $A = 4$. The softening Duffing effect is enhanced as we increase the amplitude A, as we can verify as the whole region is getting away from $\delta_\rho = 0$ to the left, i.e., negative values of δ_ρ.

The reduction of the oscillation frequency Ω_0 as we increase the amplitude A is called softening Duffing, which is expected because we have $\gamma < 0$ (a consequence of the chosen optical detuning $0 < \Delta_x < \kappa/2$). If we had chosen other detuning Δ_x, for example, $\Delta_x > \kappa/2$ or $\Delta_x < 0$, we would have $\gamma > 0$ and we would see the hardening Duffing effect, which is the shift of Ω_0 to higher frequencies. Not only this but for high enough amplitudes we can obtain larger 2 : 1 AT than 1 : 1, which is the case for $A = 3$ and $A = 4$, showing that our model still has features about high-harmonic synchronization, the same features of the experiment. To finalize this section, we conclude that each of the terms F, α, β and γ are directly proportional to the tongue width $\Delta\Omega(p, q)$ with $p = \{1, 2, 3, 4\}$ and $q = 1$, respectively, as we can see at the denumerator of Equation (47), i.e.,

$$\begin{cases} \Delta\Omega(1,1) = 2 \times \left(\frac{F}{2A} - \frac{3\beta A}{8}\right) \approx \frac{F}{A} \propto F \propto F_0 \\ \Delta\Omega(2,1) = 2 \times \left(\frac{\alpha}{4} + \frac{\gamma A^2}{4}\right) \approx \frac{\alpha}{2} \propto \alpha \propto F_1 \\ \Delta\Omega(3,1) = 2 \times \frac{\beta A}{8} \propto \beta \propto F_2 \\ \Delta\Omega(4,1) = 2 \times \frac{\gamma A^2}{16} \propto \gamma \propto F_3 \end{cases} \tag{48}$$

and these are the semi analytical expressions for the tongue width of each harmonic, which we could engineer it to achieve wider Arnold tongues for different harmonics choosing different F, α, β and γ as we design the geometry and the materials of our optomechanical cavity.

Supplementary Note 7.

Sidebands around the carrier at the synchronized region. To explain the sidebands around the synchronization region we will first linearize Equation (39) and Equation (40) expanding $A(T)$ and $\Phi(T)$ as

$$A(T) = \overline{A} + \delta A(T) \qquad \text{and} \qquad \Phi(T) = \overline{\Phi} + \delta\Phi(T), \tag{49}$$

and then diagonalize the linear part of the system

$$\frac{d}{dT}\begin{pmatrix} \delta A \\ \delta\Phi \end{pmatrix} = \begin{pmatrix} H_{AA} & H_{A\Phi} \\ H_{\Phi A} & H_{\Phi\Phi} \end{pmatrix} \begin{pmatrix} \delta A \\ \delta\Phi \end{pmatrix}, \tag{50}$$

in which the actual form of H_{AA}, $H_{A\Phi}$, $H_{\Phi A}$ and $H_{\Phi\Phi}$ are too big to be shown and not important for our present analysis. The eigenvalues of this system give us the first order correction in frequency and damping of our oscillator. The evolution of this system is

$$\begin{pmatrix}\delta A(T)\\ \delta\Phi(T)\end{pmatrix} = \begin{pmatrix}\delta A_+\\ \delta\Phi_+\end{pmatrix} e^{\left(\lambda^+_{\rm Re}+i\lambda^+_{\rm Im}\right)T} + \begin{pmatrix}\delta A_-\\ \delta\Phi_-\end{pmatrix} e^{\left(\lambda^-_{\rm Re}+i\lambda^-_{\rm Im}\right)T}, \tag{51}$$

where $\lambda^\pm = \lambda^\pm_{\rm Re} + i\lambda^\pm_{\rm Im}$ are the eigenvalues. Using these in $y(T)$ we can now search for sidebands, i.e.,

$$y(T) = A(T)\sin\left[T+\Phi(T)\right] \approx \frac{1}{2i}\left(\overline{A}+\delta A\right)\left[\left(1+i\delta\Phi\right)e^{iT}e^{i\overline{\Phi}} - \left(1-i\delta\Phi\right)e^{-iT}e^{-i\overline{\Phi}}\right] \approx$$
$$\approx \overline{A}\sin\left(T+\overline{\Phi}\right) + \delta A(T)\sin\left(T+\overline{\Phi}\right) + \overline{A}\delta\Phi(T)\cos\left(T+\overline{\Phi}\right). \tag{52}$$

It is evident now that our oscillator has more than one single frequency because of the product $\delta A(T)$ with the sine function and also because of the product $\delta\Phi(T)$ with the cosine. The frequencies $\Omega^\pm_{\rm SB}$ and the linewidths $\Gamma^\pm_{\rm SB}$ of these new sidebands are given by

$$\Omega^\pm_{\rm SB} = \frac{\lambda^\pm_{\rm Im}}{L_{\tilde{t}}} \qquad \text{and} \qquad \Gamma^\pm_{\rm SB} = \frac{\lambda^\pm_{\rm Re}}{L_{\tilde{t}}}, \tag{53}$$

and the graph of these using our semi-analytical model for the case $\rho = 1$ is shown in Supplementary Figure 15, which has an excellent agreement with the experimental data in both frequency and linewidth, showing us that these sidebands are indeed a coupling between phase and amplitude of the mechanical oscillator.

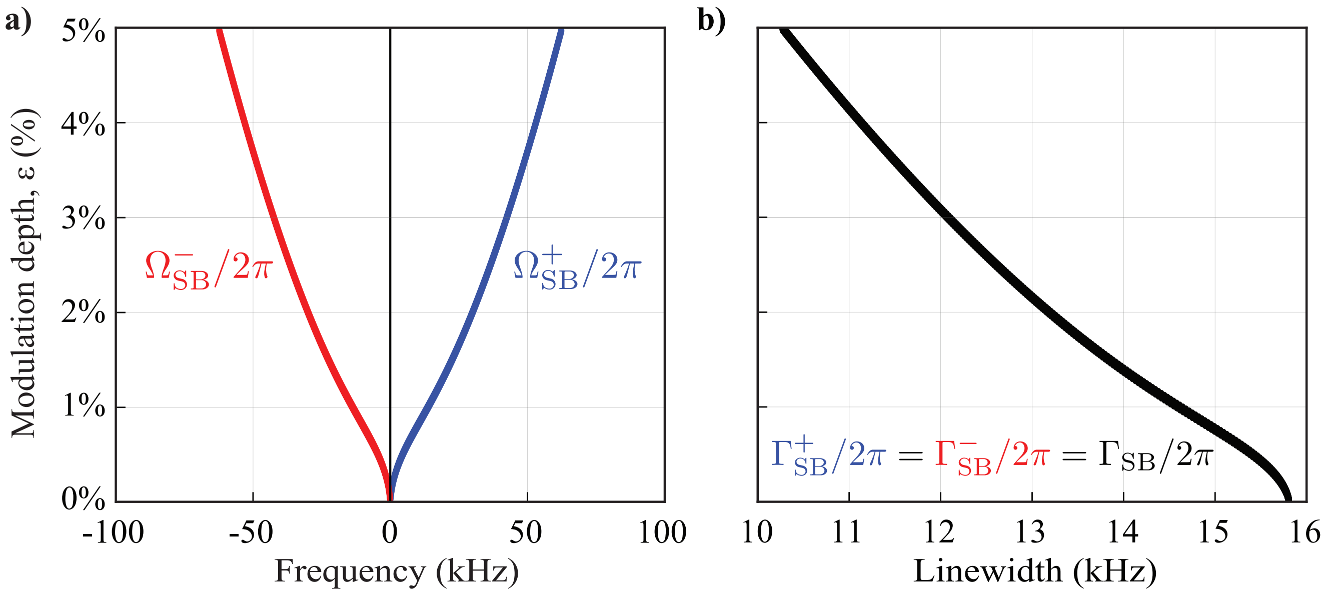

Supplementary Figure 15. **a)** Detuning from $\Omega_0/2\pi$ showing both sidebands emerging as we increase the modulation depth; **b)** Linewidth of these sidebands.

Supplementary Note 8.

1:1 Arnold Tongue Cusp Deviation. The purple cusp simulation in Fig.2 (d3) from the main text (also shown again in Supplementary Figure 16(a)), which is not present in the experimental data, was found to be a bifurcation in the amplitude dynamics. To show this we must investigate Equation (39) in more detail:

$$\left\langle\frac{dA}{dT}\right\rangle_\phi = \frac{\mu A}{2}\left(1-\frac{\sigma A^2}{4}\right) +$$
$$+ \frac{\varepsilon\sin(\pi\omega)}{\pi}\left(\frac{\omega\left[F\left(\omega^2-9\right)+2\beta A^2\right]\sin\left[(\pi-\Phi)\omega\right]}{\left(\omega^2-9\right)\left(\omega^2-1\right)} - \frac{A\left[\alpha\left(\omega^2-16\right)-6\gamma A^2\right]\cos\left[(\pi-\Phi)\omega\right]}{\left(\omega^2-16\right)\left(\omega^2-4\right)}\right). \tag{54}$$

We are searching for bifurcations around the 1 : 1 Arnold tongue, so it's natural to expand such equation around $\omega = 1$, identically as we've done with the phase Φ in Equation (45), i.e.,

$$\left\langle \frac{dA}{dT} \right\rangle_\phi = \frac{\mu A}{2}\left(1 - \frac{\sigma A^2}{4}\right) - \varepsilon\left(\frac{F}{2} - \frac{\beta A^2}{8}\right)\sin\left[\Phi(T)\right]. \tag{55}$$

The fixed points of this equation are such that $\dot{A} = \dot{\Phi} = 0$. As the bifurcation occurs at the boundary of the Arnold tongue, we will assume a fixed phase Φ of $\pi/2$ and $3\pi/2$ (which, respectively, minimize and maximize $\sin\Phi$), such that the equation that we must solve for A is

$$0 = \frac{\mu A}{2}\left(1 - \frac{\sigma A^2}{4}\right) \pm \varepsilon\left(\frac{F}{2} - \frac{\beta A^2}{8}\right). \tag{56}$$

We know that every n-degree polynomial has n complex roots, so we can plot the absolute value of each of these roots as a function of ε (we will use the same μ, σ, F, and β parameters from the simulation), and the result is shown in Supplementary Figure 16(b)

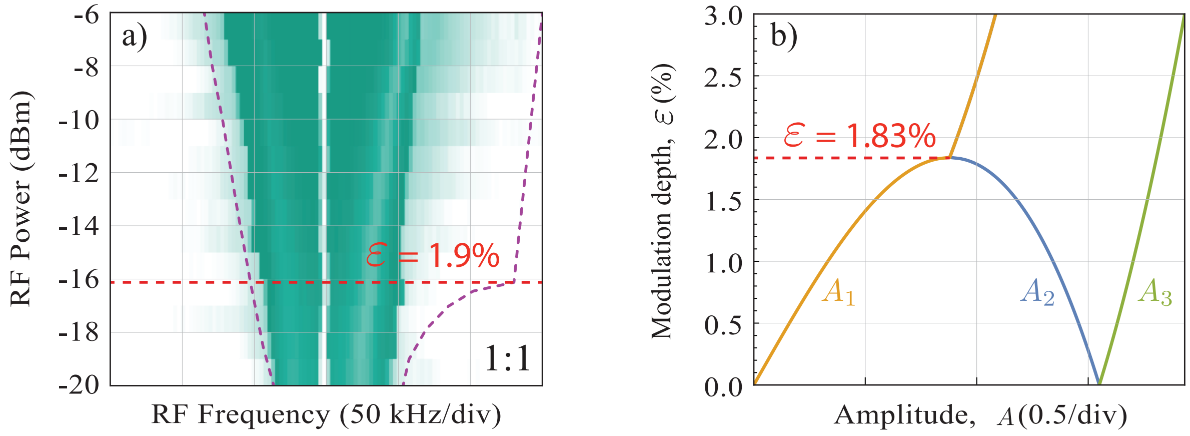

Supplementary Figure 16. **a)** Experimental 1 : 1 Arnold tongue with its simulated boundary in purple. The cusp occurs around -16 dBm, which corresponds to $\varepsilon = 1.9\%$; **b)** Amplitude evolution of each A_n root from Equation (56) as the modulation depth ε increases.

As we see, two of the roots (A_1 and A_2) of Equation (56) become degenerate (the orange and blue curves of Supplementary Figure 16(b)) for a modulation depth around $\varepsilon = 1.83\%$. The cusp that we have at the Supplementary Figure 16(a) happens around -16 dBm, which corresponds to a modulation depth of 1.9%. We must not forget that Equation (56) is one of the final steps of our semi-analytical analysis, and many simplifications were involved. Nevertheless, the purple curve in Supplementary Figure 16 was obtained using the full optomechanical model, and a difference of only 0.07% in ε is observed. Similar cusps were observed in other injection-locking experiments [8]. A more detailed analysis and more experimental data should be gathered to understand why our experiment does not exhibit this feature.

SUPPLEMENTARY REFERENCES

[1] Markus Aspelmeyer, Tobias J. Kippenberg, and Florian Marquardt. Cavity optomechanics. *Reviews of Modern Physics*, 86(4):1391–1452, 12 2014.

[2] King Yan Fong, Menno Poot, Xu Han, and Hong X. Tang. Phase noise of self-sustained optomechanical oscillators. *Phys. Rev. A*, 90:023825, Aug 2014.

[3] M. L. Gorodetksy, A. Schliesser, G. Anetsberger, S. Deleglise, and T. J. Kippenberg. Determination of the vacuum optomechanical coupling rate using frequency noise calibration. *Opt. Express*, 18(22):23236–23246, Oct 2010.

[4] E.A. Jackson. *Perspectives of Nonlinear Dynamics: Volume 1*. Perspectives of Nonlinear Dynamics. Cambridge University Press, 1989.

[5] Cijy Mathai, Sunil A. Bhave, and Siddharth Tallur. Modeling the colors of phase noise in optomechanical oscillators. *OSA Continuum*, 2(7):2253–2259, Jul 2019.

[6] Shreyas Y. Shah, Mian Zhang, Richard Rand, and Michal Lipson. Master-slave locking of optomechanical oscillators over a long distance. *Phys. Rev. Lett.*, 114:113602, Mar 2015.

[7] Keren Shlomi, D. Yuvaraj, Ilya Baskin, Oren Suchoi, Roni Winik, and Eyal Buks. Synchronization in an optomechanical cavity. *Phys. Rev. E*, 91:032910, Mar 2015.

[8] J. Simonet, M. Warden, and E. Brun. Locking and arnold tongues in an infinite-dimensional system: The nuclear magnetic resonance laser with delayed feedback. *Phys. Rev. E*, 50:3383–3391, Nov 1994.

[9] Mian Zhang, Gustavo Luiz, Shreyas Shah, Gustavo Wiederhecker, and Michal Lipson. Eliminating anchor loss in optomechanical resonators using elastic wave interference. *Applied Physics Letters*, 105(5):051904, Aug 2014.
